# Modelling aptamers with nucleic acid mimics (NAM): From sequence to three-dimensional docking

**DOI:** 10.1371/journal.pone.0264701

**Published:** 2022-03-23

**Authors:** Ricardo Oliveira, Eva Pinho, Ana Luísa Sousa, Óscar Dias, Nuno Filipe Azevedo, Carina Almeida

**Affiliations:** 1 INIAV - National Institute for Agrarian and Veterinarian Research, Rua dos Lagidos, Vairão, Vila do Conde, Portugal; 2 LEPABE - Laboratory for Process Engineering, Environment, Biotechnology and Energy, Faculty of Engineering, University of Porto, Rua Dr. Roberto Frias, Porto, Portugal; 3 Centre of Biological Engineering (CEB), University of Minho, Campus de Gualtar, Braga, Portugal; 4 LABBELS – Associate Laboratory, Braga, Guimarães, Portugal; Lehman College, UNITED STATES

## Abstract

Aptamers are single-stranded oligonucleotides, formerly evolved by Systematic Evolution of Ligands by EXponential enrichment (SELEX), that fold into functional three-dimensional structures. Such conformation is crucial for aptamers’ ability to bind to a target with high affinity and specificity. Unnatural nucleotides have been used to develop nucleic acid mimic (NAM) aptamers with increased performance, such as biological stability. Prior knowledge of aptamer-target interactions is critical for applying post-SELEX modifications with unnatural nucleotides since it can affect aptamers’ structure and performance. Here, we describe an easy-to-apply *in silico* workflow using free available software / web servers to predict the tertiary conformation of NAM, DNA and RNA aptamers, as well as the docking with the target molecule. Representative 2ʹ-O-methyl (2ʹOMe), locked nucleic acid (LNA), DNA and RNA aptamers, with experimental data deposited in Protein Data Bank, were selected to validate the workflow. All aptamers’ tertiary structure and docking models were successfully predicted with good structural similarity to the experimental data. Thus, this workflow will boost the development of aptamers, particularly NAM aptamers, by assisting in the rational modification of specific nucleotides and avoiding trial-and-error approaches.

## Introduction

Aptamers are single-stranded oligonucleotides generated by Systematic Evolution of Ligands by EXponential Enrichment (SELEX), whose functionality is strictly dependent on their tertiary structure [1]. Traditional DNA and RNA aptamers have low chemical and biological stability; but unnatural nucleotides can be used to improve their performance. Unnatural nucleotides comprise chemical modifications on the heterocyclic base or sugar-phosphate backbone of native nucleotides [2]. They can be inserted by either *de novo* SELEX (a selection process starting with a NAM (nucleic acid mimic)-based library) or post-SELEX modifications (nucleotide substitutions by unnatural nucleotides at specific positions) [3]. Although post-SELEX approaches have been widely used to enhance previously selected aptamers due to their ease of application, they are often based on trial-and-error methodologies [4, 5]. In addition, the applied modifications often alter aptamers’ tertiary structure and, consequently, affect aptamer-target binding interaction [3, 6, 7]. Thus, knowledge of the tertiary structure of aptamers and their interaction with the target molecule is crucial to successfully perform post-SELEX modifications [8]. Such information can be obtained by experimental techniques such as magnetic resonance spectroscopy (NMR) and X-ray crystallography [8, 9]. However, NMR methods are usually limited to relatively small molecules (<40 kDa) due to the complexity of the data analysis, and X-ray crystallography requires crystals to provide adequate quality diffraction data and crystal structures of aptamer-target complexes have proven to be challenging to obtain. Moreover, both techniques require specialized equipment and technicians not available in most research laboratories [10].

Alternatively, *in silico* approaches have been proposed to assist post-SELEX modifications [8], including computational tools to predict aptamer structures and thermodynamic properties, as well as aptamer-target docking models (identification of key interaction residues, structural motifs, docking structures, etc.) [11]. Thus, they might allow a tailored selection of nucleotides to be modified to improve aptamer performance, saving experimental time in the conventional trial-and-error approach [8, 11]. Some workflows have been recently published to predict aptamers’ tertiary structure, thermodynamic properties or docking models, starting from the aptamer primary structure (linear sequence), using bioinformatics tools designed for modelling nucleic acids [8, 11–17]. At the moment, there is no complete *in silico* workflow to help determine the three-dimensional conformation of aptamers and the binding to their target [17]. Software and web servers are dispersed by the internet, requiring logical assemble and careful choice of the most suitable tools.

Moreover, most computational tools have been designed for RNA; thus, having to be adapted to predict the tertiary structure of single-stranded DNA. Additionally, they are either unable to address NAM and/or require specialized computational and molecular modelling skills to use them [13, 18, 19].

Hence, to assist NAM aptamers development and boost their applicability, we propose a workflow that allows predicting the tertiary structure and interaction model with the target molecule of aptamers containing unnatural nucleotides. Moreover, the developed workflow is based on freely available software and web server without the need of specialized skills to use such tools.

## Methods

### Software workflow

A workflow, based on freely available bioinformatics tools, was developed to predict the tertiary structure of NAM, RNA and DNA aptamers (using the aptamers sequence as a starting point) and the docking model (including the identification of residues interacting with the target molecule). Several software/ web servers were assessed to establish the workflow. For the secondary structure assemble, Kinefold [20], Mfold [21], MPGAfold [22], NUPACK [23], RNAFold [24], RNAstrucure [25] and RNA2D3D [26] were evaluated. For tertiary structure prediction, 3D-DART [27], 3dRNA [28], ASSENBLE2 [29], NAMD [30], RNAComposer [31], Rosetta [32], YASARA [33] and Vfold3D [34] were tested. For inserting chemically modified nucleotides, compatible with the selected unnatural nucleotides, ASSEMBLE2 [29], BIOVIA Discovery Studio, PyMOL, and YASARA [33] were assessed. Finally for the docking model, AutoDock [35], GOLD [36], GRAMM-X [37], HDOCK [38], NPDock [39] and Patchdock [40] were used. Suitability, ease of handling, and cost were used as criteria for selecting the software/web servers to assemble the workflow.

**Fig 1:**
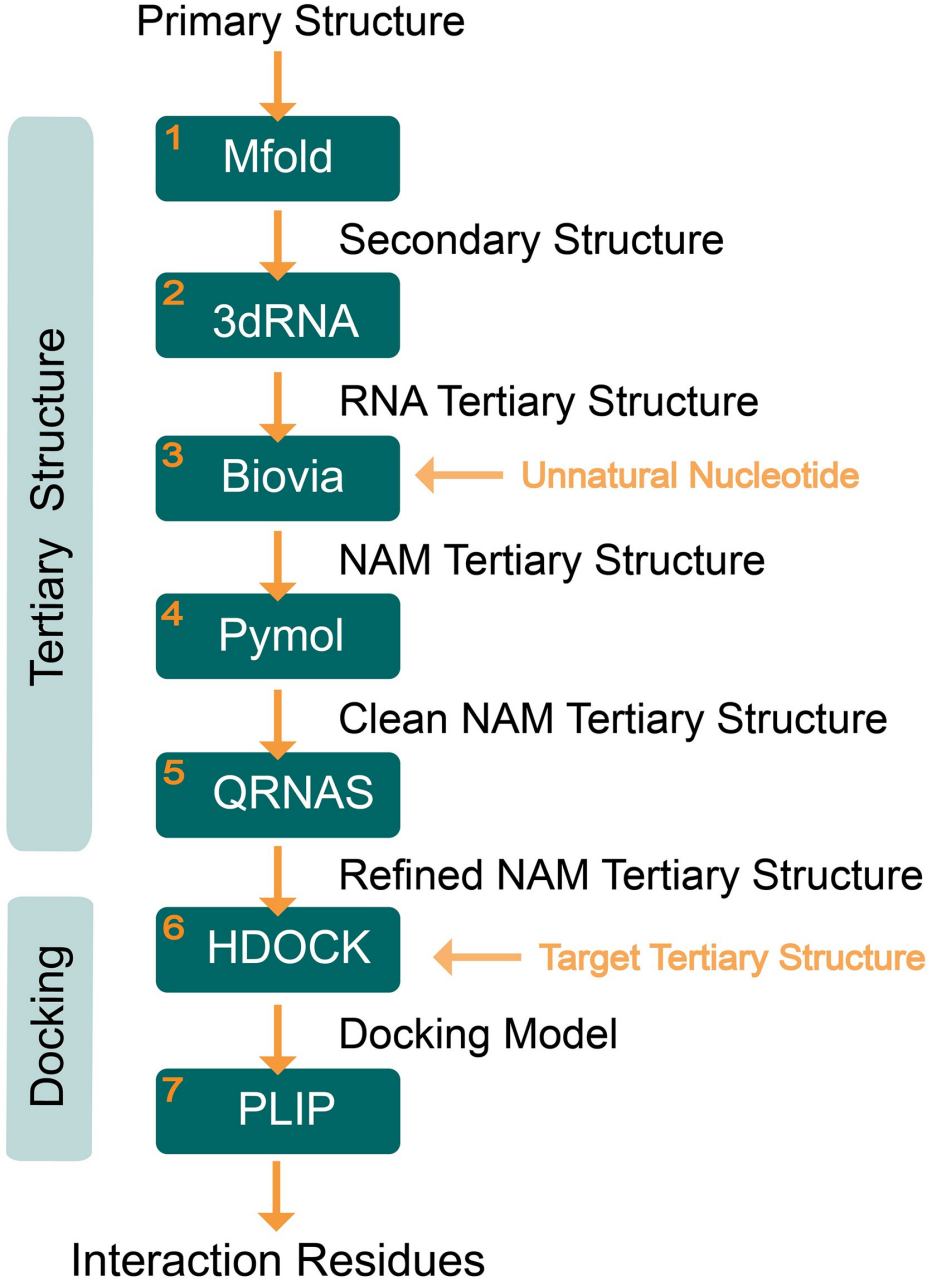
Workflow to predict the tertiary structure of NAM aptamers (using the aptamers sequence as a starting point) and the docking model (including the identification of nucleotides that interact with the target molecule). The yellow horizontal arrows are additional inputs necessary to run the workflow.

Briefly, the workflow comprises seven main steps (Fig 1): **(1)** predict the secondary structure from the nucleotide sequence using the Mfold web server (http://unafold.rna.albany.edu/?q=mfold) [21], **(2)** assemble the tertiary structure of the corresponding RNA model from the secondary structure on the 3dRNA v2.0 web server (http://biophy.hust.edu.cn/new/3dRNA) [41], **(3)** transform the tertiary RNA structure into a DNA or NAM structure through BIOVIA Discovery Studio software (v 20.1.0.19295), **(4)** add hydrogen atoms using the PyMOL software (windows version 2.4.0) since these atoms (often omitted in chemical structures) play a major rule on the stabilization of aptamers tertiary structure and interaction with the target [42], **(5)** refine the final tertiary structure on QRNAS software (Ubuntu version 0.3—Quick Refinement of Nucleic Acids 0.3) [42], **(6)** simulate the docking models through the HDOCK web server (http://hdock.phys.hust.edu.cn/) [38] and **(7)** identify the interaction residues using the Protein-Ligand Interaction Profiler (PLIP) web server (https://projects.biotec.tu-dresden.de/plip-web/plip/index) [43].

#### Step 1: Aptamers’ secondary structure

The nucleotide sequences were used as input to predict aptamers’ secondary structure based on a free energy minimization model applying the Mfold web server [21]. Regarding DNA-like sequences, the folding temperature and ionic buffer conditions (Na^+^ and Mg^2+^ concentrations) were adjusted at the "DNA Folding Form" according to the experimental selection conditions of each aptamer, while the remaining parameters were used with the default values (http://www.unafold.org/mfold/applications/dna-folding-form.php). For RNA-like sequences, the secondary structure of the aptamers was predicted with the RNA Folding Form V2.3 (http://www.unafold.org/mfold/applications/rna-folding-form-v2.php), using the folding temperature and ionic buffer conditions fixed at 1 M. For each aptamer, the most thermodynamically stable structure (lowest Gibbs free energy (ΔG) value) was selected, and the corresponding Vienna output file (dot-bracket notation -. ct file) saved.

#### Step 2: Tertiary structures of the equivalent RNA aptamers

Aptamers tertiary structure was assembled using the fully automatic 3dRNA v2.0 web server [28]. The nucleotide sequence and the respective dot-bracket notation (Vienna file), obtained in step 1, were used as input. As the 3dRNA v2.0 was developed for RNA structures, the nucleotide thymine (T) was replaced by uracil (U) for DNA-like aptamers. The *Procedure Optimize*, *5 predictions*, *3dRNA-Lib2* and *Minimization* were used as advanced options. The tertiary structures with the lowest score (better statistical model) were saved as a Protein Data Bank (pdb) file [41, 44].

#### Step 3: Mutation of RNA structures to DNA or NAM structures

The Biovia Discovery Studio Visualizer software was used convert RNA tertiary structures to DNA or NAM structures (when applicable). Conversion to DNA or NAM was performed by changing the nitrogenous bases from uracil to thymine, the pentoses from ribose to deoxyribose, or inserting the corresponding unnatural nucleotide.

#### Steps 4 and 5: Refine the final tertiary structures of aptamers

The tertiary structures were imported into PyMOL to add hydrogen atoms, often omitted from the simulated structures but with an important rule on the tertiary and molecular docking. The structures were then saved as pdb files and, finally, optimized by the steepest descent energy minimization method (100 000 steps) using the automated QRNAS software. This tool performs a fine-grained refinement of nucleic acid tertiary structures generated in previous steps by simplified methods that use pre-existing structures in databases as templates to develop a comparative model with low- to medium-resolution. QRNAS is an improved version of the AMBER simulation method that includes energy minimization of hydrogen bonds, base pairs, backbone irregularities, and custom restraints [42].

#### Step 6: Simulation of the interaction between aptamers and their targets

The target molecule was isolated by removing the experimentally resolved aptamer structure, ions or water molecules and adding the omitted hydrogens atoms using PyMOL software (clean structure) from the file downloaded from the database *Protein Data Bank in Europe* (PDBe, https://www.ebi.ac.uk/pdbe/). This step aims to normalize the effect of the solvent and ensure proper hydration of the functional groups [45]. The docking simulation was performed on HDOCK web server [38], using the tertiary structure of each aptamer (Step 5 pdb file) and target molecule (clean structure) as input.

#### Step 7: Identification of interactions between the aptamer and the target

The best docking model (lowest docking energy score) was selected and compared with the experimentally resolved aptamer-protein structures using PyMOL. The interacting residues between the aptamers and the targets were identified with the PLIP web server, using the docking model obtained on step 6 as input.

### Workflow validation

A search for experimentally resolved structures of NAM, RNA and DNA, aptamers complexed with a protein molecule, was performed in the PDBe (https://www.ebi.ac.uk/pdbe/). From the three independent searches—using the terms "nucleic acid mimic aptamer", "DNA aptamer", and "RNA aptamer"—925 structures were identified. The structures were then individually reviewed, and the following exclusion criteria were applied: 1) lack of experimental resolved aptamer-protein complex; 2) inclusion of triplexes, duplexes, G-quadruplexes or kissing complexes (due to software/web server limitations); and 3) inclusion of other types of unnatural nucleotides besides LNA (locked nucleic acid) or 2ʹ-O-methyl (2ʹOMe) nucleotides. The version of the software (BIOVIA) used to include unnatural nucleotides in the aptamer sequence only accepts 2ʹOMe and LNA.

After the first five steps of the workflow, the predicted tertiary structure of the aptamers was compared with experimental data from PDBe using RMSD (root mean standard deviation) as a quantitative parameter. For this, the files were open on the PyMOL software, and the RMSD was obtained with the align plugin, assuming five outlier rejection cycles and a cut-off of 2.0. The *in silico* docking models were evaluated based on the superposition with experimental data by RMSD and the number of shared contact nucleotides.

## Results and discussion

The aptamers’ performance is strictly linked to their conformational structure since aptamers’ functionality relies on the physical fitting between the oligonucleotide and the target [46]. In contrast, their structure is determined by the nucleotide sequence and how that specific combination of nucleotides assembles into a three-dimensional conformation. Hence, the prediction of the tertiary structure is vital to identify residues responsible for the aptamer conformation and aptamer-target interaction.

Computational tools are an asset for developing and optimizing aptamers by avoiding time-consuming experimental procedures, especially when aiming post-SELEX modifications. Although there are several software and web servers for application of natural nucleic acids, no works have been published using such tools with NAMs. Thus, the authors compiled a workflow with free software and web servers (Fig 1) able to build the tertiary structure of NAM aptamers, besides RNA and DNA aptamers, using the nucleotide sequences as a starting point. Also, the workflow can simulate the molecular docking between the aptamers and their protein targets and identifying the physical-chemical interactions. The combination of these functionalities allows to predict the structural effect of modifications with unnatural nucleotides on the aptamers tertiary structure and docking model.

### Selection of aptamer-target models

After an extensive analysis, two NAM aptamers containing 2ʹOMe nucleotides (PDB ID: 5D3G and 5HRT), a DNA (PDB ID: 6U82) and an RNA (PDB ID: 4PDB), were selected as study models to validate the workflow (Table 1). No experimentally resolved structure of LNA aptamers complexed to a protein-ligand was found in PDBe. Thus, an exception was made for an LNA aptamer (PDB ID: 2PN9) whose target is a nucleic acid (kissing complex). The LNA aptamer was used only as a model for the first phase of the workflow to predict the tertiary structure and compared with the experimental structure deposited on PDBe.

**Table 1:** Aptamer models selected to validate the assembled workflow. The position of the modified residues is identified in the sequence with "m" preceding the respective nucleotide. The SELEX conditions were used as input on step 1 of the workflow.

PDBe ID	nt	Sequence	Aptamer type	Target	SELEX Conditions	Ref
**5D3G**	38	TAATACmCCmCCCCTTCGGTGCTTTG CACCGAAGGGGGGG	2ʹOMe	HIV-1 Reverse Transcriptase	25°C	[47]
					0 mM Na^+^	
					2 mM Mg^2+^	
**5HRT**	34	CCTGGAmCGmGAACCmAmGAATmAm CTTTTGGTCTCCmAmGmG	2ʹOMe	Autotaxin	25°C	[48]
					145 mM Na^+^	
					0.8 mM Mg^2+^	
**2PN9**	16	CACGGUCCmCmAGACGUG	LNA	TAR RNA element of HIV-1	23°C	[49]
					0 mM Na^+^	
					0 mM Mg^2+^	
**6U82**	38	GCTAATCTAATCAACCGCAGGT TGATTAGCCCATTAGC	DNA	Double homeobox protein 4	20°C	[50]
					150 mM Na^+^	
					5 mM Mg^2+^	
**4PDB**	38	GGGAUGCUCAGUGAUCCUUCGG GAUAUCAGGGCAUCCC	RNA	*B*. *anthracis* S8 protein	4°C	[51]
					100 mM Na^+^	
					5 mM Mg^2+^	

### Prediction of aptamers’ tertiary structure

**Fig 2:**
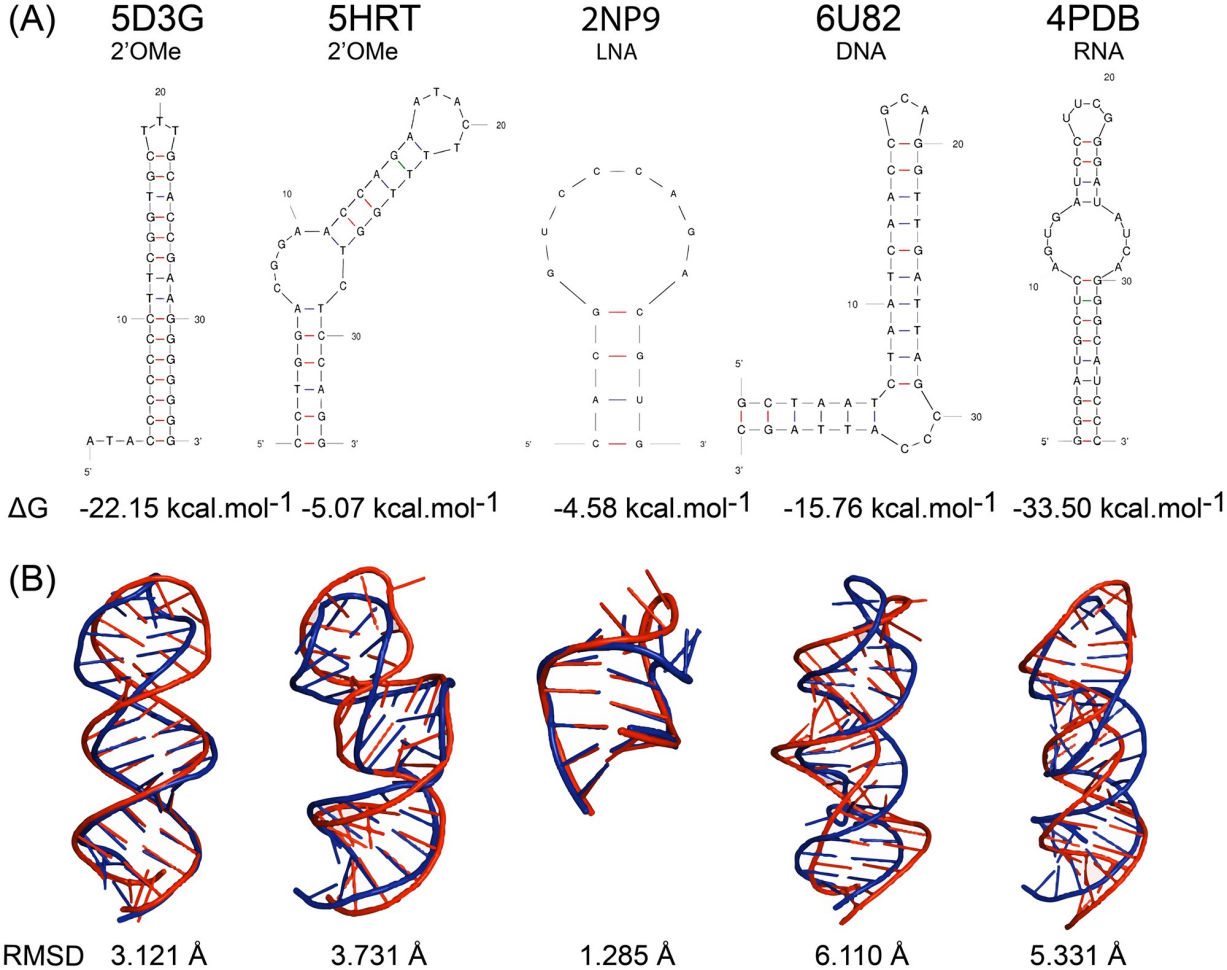
Aptamers’ secondary (A) and tertiary (B) structure obtained after executing the first five steps of the workflow (Mfold, 3dRNA, Biovia, PyMOL and QRNAS). (A) The secondary structures obtained by Mfold and the Gibbs free energy (ΔG). (B) The overlap of the predicted tertiary structures (in red) and the corresponding experimentally resolved structures downloaded from the PDBe (in blue), and the RMSD values.

The secondary and tertiary structures of NAM, RNA and DNA aptamers were successfully built (Fig 2) by applying the assembled workflow, using the nucleotide sequences as a starting point. Regarding the secondary structure, the ΔG of the selected structures ranged between -33.50 kcal.mol^-1^ (4PDB) and 4.58 kcal.mol^-1^ (2PN9) (Fig 2A). This parameter is influenced not only by the nucleotide composition, but also by its folding pattern and the conditions in which it was determined [21]. Based on Fig 2A, the five aptamers showed similar secondary structures with stems, internal loops and hairpin loops. Accordingly, the tertiary structures were also quite identical (Fig 2B). Each predicted tertiary structure was aligned with the experimental structure deposited in the PDBe database. The RMSD value, a measure of the average distance between the atoms of superimposed macromolecules [52], was calculated using the PyMOL alignment plugin to measure the degree of similarity between predicted and experimentally resolved tertiary structures.

Aptamers structures obtained *in silico* were structurally identical to the experimental data (Fig 2(B)) with RMSD values ranging from 1.285 Å (2PN9) to 6.110 Å (6U82). The LNA aptamer (2PN9) had a simpler three-dimensional structure mainly due to its small size (16 nucleotides), probably justifying its lower RMSD. Similar RMSD values were reported for DNA aptamers and their experimentally deposited structures by other *in silico* workflows [13, 19].

It is important to highlight that the aptamers’ tertiary structures were predicted without considering the unnatural nucleotides since the web server are only able to use natural nucleotides. For the secondary structure prediction (Step 1), no significant interference of unnatural nucleotides with modifications on the sugar molecule, such as 2ʹOMe or LNA, is expected since it is determined by intramolecular Watson-Crick complementary base pairing [12]. However, the tertiary structure is dependent on geometrical and steric constraints imposed by the nucleotides that compose aptamers [53]. Thus, the fine-grained refinement of the aptamers (Step 4 and 5) was vital to accommodate the modifications inserted after the tertiary structure prediction (Step 3) to minimize the imperfections in the prediction of NAM and DNA structures in the form of RNA (Step 2).

In the present work, only aptamers with 2ʹOMe or LNA modifications were used since the bioinformatic tool applied to change the tertiary structure from RNA to NAM was only capable of working with these two type of unnatural nucleotides. Other tools, such as Assemble or RNA2D3D, may allow the use of other unnatural nucleotides. Although, they require manual molecular interactions inputs that are usually unknown due to the lack of experimentally resolved structures of NAM aptamers. In fact, one major limitation when working with NAM aptamers is the absence of experimental data to feed the databases used by the bioinformatics tools. Moreover, from the authors’ experience, most of the available tools that could work with unnatural nucleotides involve, besides advanced computational skills, access to online databases (not always available) and are often limited to small size oligonucleotides [9, 13, 17, 54].

### Simulation of the interaction between aptamers and their targets

**Fig 3:**
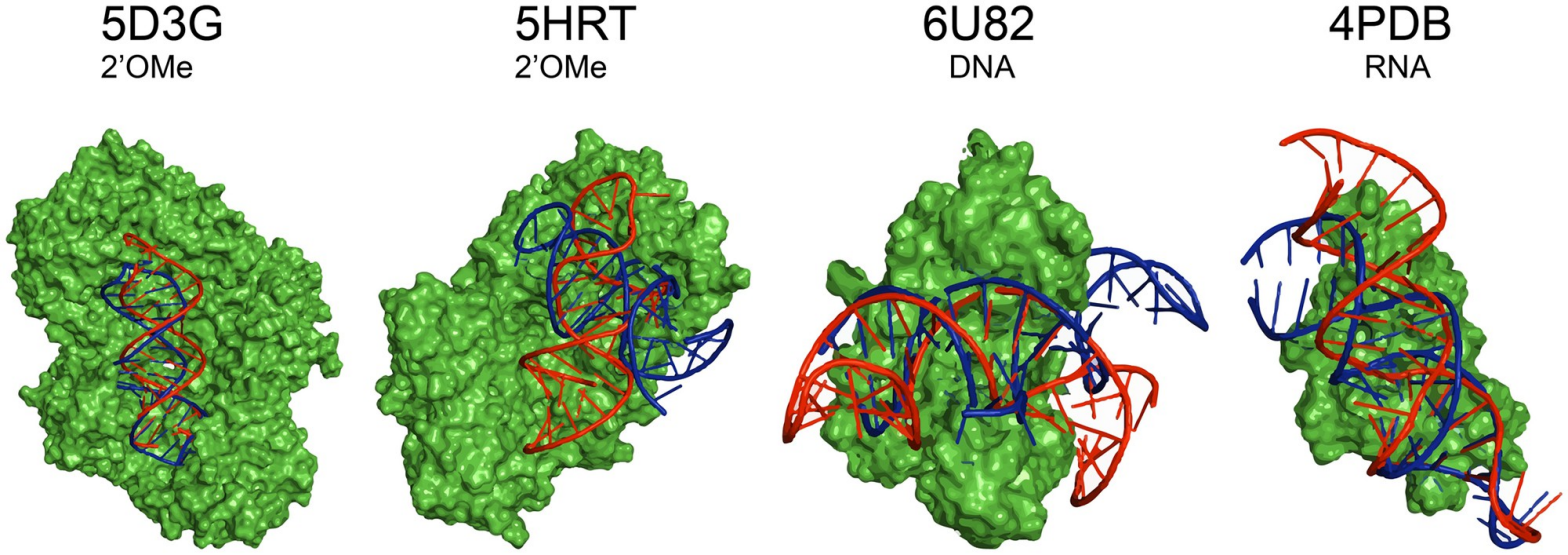
Comparison of the docking models deposited experimentally (in blue) in the PDBe database and the *in silico* docking models predicted through the described workflow (in red) for the 2ʹOMe (5D3G and 5HRT), DNA (5HRU) and RNA (6SY4) aptamers. The target molecules (in green) were isolated from the aptamer-target complexes determined experimentally using the PyMOL software and used as a receptor in the docking prediction.

The molecular docking of the aptamers with the target molecules was simulated on the HDOCK web server [38] using the tertiary structures obtained after step 6 as input. *In silico* docking resorts to a bioinformatic process that sample all possible binding positions of the aptamer to the target. Then, the assembled docking models are sorted by a scoring function. In the case of HDOCK, the score classification takes into account the shape complementarity as well as the potential intermolecular interactions involved in the nucleic acid-target stabilization [38, 55]. Although several possible docking models were obtained, only the model with the best docking score for each aptamer was used for further analysis (Fig 3). As mentioned before, the LNA aptamer was not included in this step since its target is a nucleic acid (kissing complex), and the applied bioinformatics tool can only predict nucleic acid-protein target docking models [38].

Comparing the *in silico* molecular docking and the experimentally deposited complexes, a similarity of the aptamer-target interacting regions was verified for the four aptamer models used. The docking model obtained for 5D3G was the most identical to the experimental data, with both aptamers (*in silico* and experimental) showing the same orientation within the target (Fig 3). Similar behavior was observed for the RNA aptamer (4PDB), where both tertiary structures interacted with the target with the same orientation. For the aptamers 5HRT and 6U82, although the docking occurs in the same target and aptamer region, *in silico* aptamers orientation differed from the experimental data.

**Fig 4:**
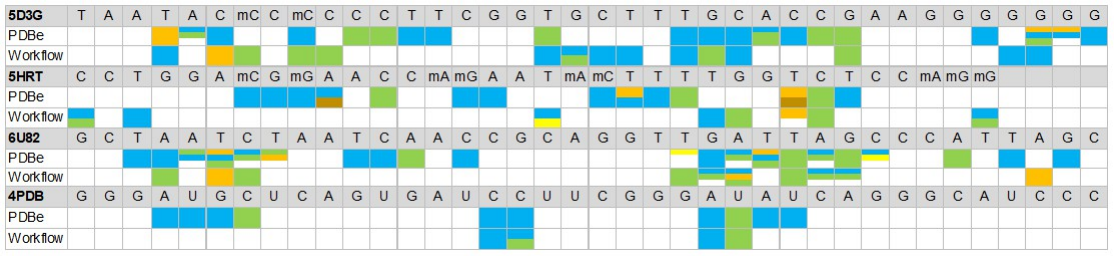
Identification of aptamer-target contact nucleotides and type of non-covalent interaction involved in the stabilization of the complexes for *in silico* (workflow) and experimental data (PDBe) docking. Hydrophobic Interactions (orange), Hydrogen Bonds (blue), Salt Bridges (green), π-Stacking (brown) and π-Cation Interactions (yellow).

Based on the best molecular docking models, the non-covalent interactions between the target molecules and aptamers were also determined (Fig 4) using the PLIP web server [43]. This computational tool allowed identifying residues and the type of interactions between the aptamer and the target protein. The interaction report identifies the chemical group involved in the interaction, improving the knowledge on the mechanisms involved in stabilizing the aptamer-target interaction. Assuming the interacting residues of the experimentally resolved aptamer-target complexes as a reference, the 2ʹOMe aptamers (5D3G and 5HRT) present 9/20 (3 residues matched the type of non-covalent interactions) and 2/14 (1 residue correspond to the type of non-covalent interactions) coincident interacting residues, respectively. DNA aptamers have 9/21 coincident interacting residues (2 residues matching the type of non-covalent interactions), and the RNA aptamer has 4/10 (3 residues are full matched with the type of non-covalent interactions).

Comparison between the experimentally deposited data and the *in silico* simulations reveals a low correspondence of interacting residues. This observation is, in part, expected since, despite the structural similarity of tertiary structures, small differences can have a great influence on the shape-based docking and, consequently, on the intermolecular interactions between the aptamer and the target molecule. Even so, a greater number of corresponding interacting residues was observed for the 5D3G model, which presents better predictions of tertiary structure and docking region.

Moreover, the aptamer’s (deoxy)ribose-phosphodiester backbone has high flexibility with a total of six torsional angles/flexible bonds, thus enabling a wide variety of tertiary structures with distinct degrees of energetic stability [55]. Hence, different conformations of the same aptamer sequence can coexist in solution, with various degrees of affinity (or, even, no affinity) to the target. The fact is that the conformation with a higher affinity to the target is not necessarily the most stable/ energetic favorable. Thus, conformations with lower stability but higher affinity for the target can be experimentally favorable at the expense of the most stable structures *in silico*. The compiled workflow uses the predicted structures (secondary, tertiary and molecular docking models) ranked by the bioinformatics tools as the most likely statistically, i.e., more energetic favorable, to simplify analysis. Also, ions and temperature play a key role in experimental conformation, and some conditions might be challenging to predict by computational tools [56]. In this workflow, environmental conditions (i.e., temperature and ions concentration) are only controllable to a certain degree in the secondary structure prediction (Step 1). This may justify the differences obtained from the *in silico* structures and the experimental data. The selected in silico structures might not correspond to the ones with higher affinity under experimental conditions. A deeper analysis of all possible structures on each step may increase the accuracy of the *in silico* prediction, despite being more time consuming and laborious. As an example, a supplementary analysis was carried to the 2ʹOMe aptamers (PDB ID: 5D3G and 5HRT) by performing variations on the simulation conditions: (i) environmental conditions (i.e., Na^+^/Mg^2+^ conditions and temperature, Step 1); (ii) using a tertiary structure with a lower score (Step 2), and (iii) not carrying out the mutation of the structures with the unnatural nucleotides (Step 3) (S1, S2 and S3 Figs, respectively). It was observed that the RMSD values of the predicted tertiary structures were higher for all alternative conditions and the docking with the target protein deviated from the experimentally determined data. These structural differences were reflected in the lower correspondence of the interacting residues between the simulated models and the experimental data deposited in the PDBe database (S4 Fig).

Despite the above-mentioned limitations, the developed workflow proved to be an important tool for predicting aptamers tertiary structures (including NAM aptamers), as well as aptamers-protein target docking. It also serves as a tool for an initial analysis of the possible effect of post-SELEX modifications in aptamers conformation and interactions with the target. With this valuable information, it is possible to identify residues in different motifs of the aptamer for further substitutions with unnatural nucleotides, such as 2ʹOMe and LNA, which do not compromise the aptamer-target docking model. This allows a rational substitution of nucleotides and a suitable in silico platform to guide further benchwork.

## Conclusions

Applying unnatural nucleotides is an essential evolution of aptamers, considering the limitations in the chemical and biological stability of traditional DNA and RNA aptamers. The development of this type of nucleic acid lacks intuitive computational tools to assist decisions involving structural predictions. Moreover, from the authors’ knowledge, no workflow to predict NAM aptamer’s tertiary structure and their binding to a target molecule has been published.

Here we present a simple and easy-to-apply workflow for researchers without deep computer experience to predict the tertiary structure and the molecular docking with the target of aptamers containing unnatural nucleotides.

Although the *in silico* data generated by the workflow correlated well to the experimental data, the proposed workflow has limitations on the type of unnatural nucleotides. However, with the increasing applicability of this type of computational approach, an improvement of these tools regarding the use of more types of unnatural nucleotides is expected. Also, a broad adoption of these tools for aptamer-target interactions, associated with the experimental validation of the *in silico* data, will serve to feed and perfect the algorithms of these bioinformatics tools.

## Supporting information

S1 Fig Effect of changing the environmental conditions (Step 1) on 2ʹOMe (PDB ID: 5D3G and 5HRT) aptamers’ secondary (A), tertiary (B) structure and molecular docking model (C) simulation. For aptamer 5D3G, the secondary structure was predicted at 37°C, 100 mM Na^+^ and 1 mM Mg^2+^. Regarding, 5HRT the following conditions were used: 37°C, 50 mM Na^+^ and 2 mM Mg^2+^. (A) The secondary structures obtained by Mfold and the Gibbs free energy (ΔG). (B) The overlap of the predicted tertiary structures (in red) and the corresponding experimentally resolved structures downloaded from the PDBe (in blue), and the RMSD values. (C) Molecular docking models deposited experimentally (in blue) in the PDBe database and the *in silico* docking models predicted through the described workflow (in red). The target molecules (in green) were isolated from the aptamer-target complexes determined experimentally using the PyMOL software and used as a receptor in the molecular docking prediction.
(TIF)

S2 Fig Prediction of 2ʹOMe (PDB ID: 5D3G and 5HRT) aptamers’ secondary (A), tertiary (B) structure and docking model (C) by selecting a tertiary structure with a lower score (Step 2). (A) The secondary structures obtained by Mfold and the Gibbs free energy (ΔG) using the corresponding environmental conditions. (B) The overlap of the predicted tertiary structures (in red) and the corresponding experimentally resolved structures downloaded from the PDBe (in blue), and the RMSD values. (C) Docking models deposited experimentally (in blue) in the PDBe database and the *in silico* docking models predicted through the described workflow (in red). The target molecules (in green) were isolated from the aptamer-target complexes determined experimentally using the PyMOL software and used as a receptor in the docking prediction.
(TIF)

S3 Fig Prediction of 2ʹOMe (PDB ID: 5D3G and 5HRT) aptamers’ secondary (A), tertiary (B) structure and docking model (C) without mutation of the unnatural nucleotides (Step 3). (A) The secondary structures obtained by Mfold and the Gibbs free energy (ΔG) using the corresponding environmental conditions. (B) The overlap of the predicted tertiary structures (in red) and the corresponding experimentally resolved structures downloaded from the PDBe (in blue), and the RMSD values. (C) Docking models deposited experimentally (in blue) in the PDBe database and the *in silico* docking models predicted through the described workflow (in red). The target molecules (in green) were isolated from the aptamer-target complexes determined experimentally using the PyMOL software and used as a receptor in the docking prediction.
(TIF)

S4 Fig Identification of aptamer-target contact nucleotides and type of non-covalent interaction in the *in silico* simulations performed with alternative conditions for the 2ʹOMe aptamers. Hydrophobic Interactions (orange), Hydrogen Bonds (blue), Salt Bridges (green), π-Stacking (purple) and π-Cation Interactions (yellow).
(TIF)

S1 File Input files of all steps of the proposed workflow for all tested aptamer models. (RAR)
