# Supplementary figures and images for: Modelling aptamers with nucleic acid mimics (NAM): From sequence to three-dimensional docking
Source: PLoS One. 2022 Mar 23;17(3):e0264701. doi: 10.1371/journal.pone.0264701 (PMC8942228; doi:10.1371/journal.pone.0264701)

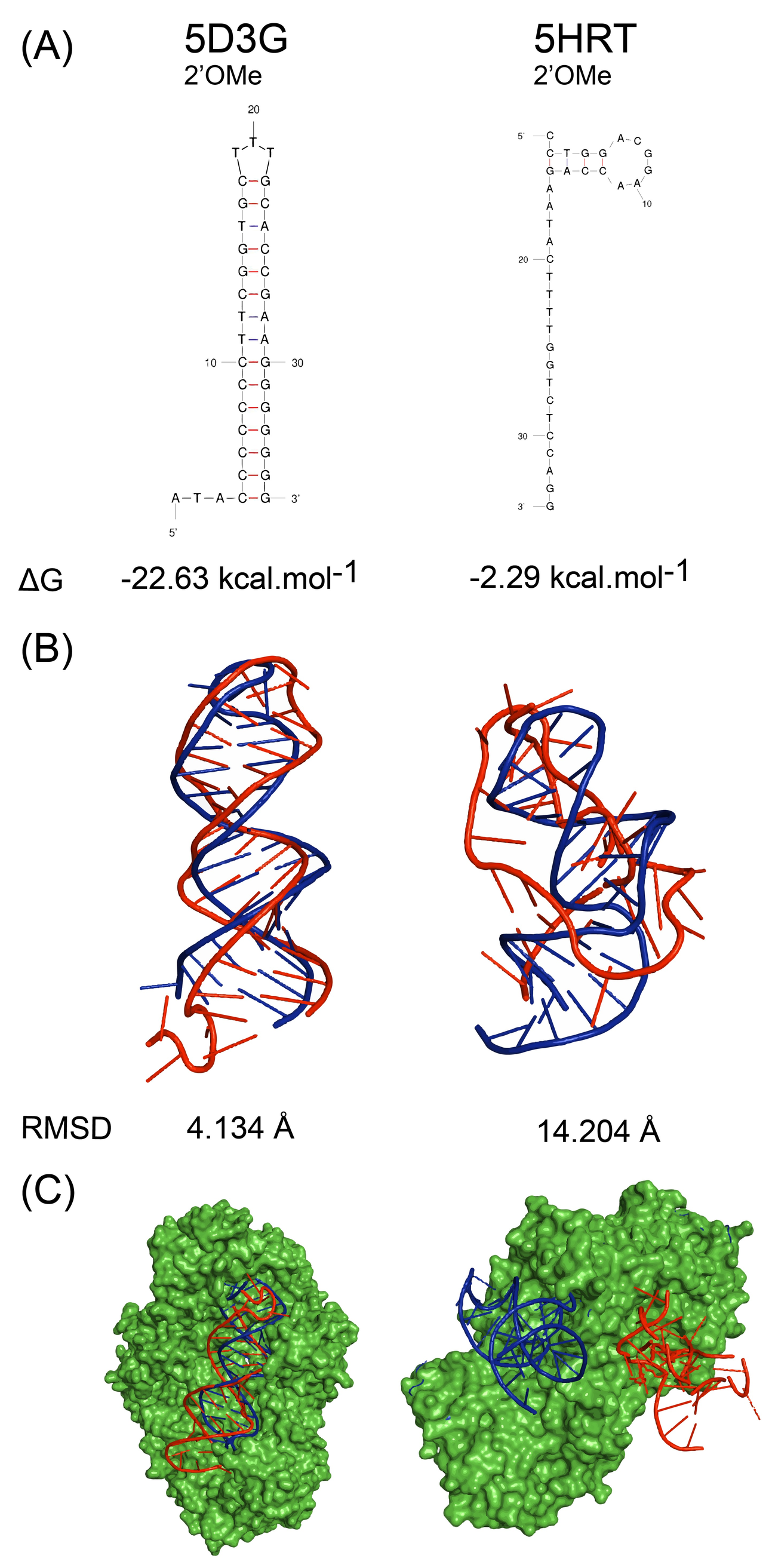

Supplement: S1 Fig — For aptamer 5D3G, the secondary structure was predicted at 37°C, 100 mM Na+ and 1 mM Mg2+. Regarding, 5HRT the following conditions were used: 37°C, 50 mM Na+ and 2 mM Mg2+. (A) The secondary structures obtained by Mfold and the Gibbs free energy (ΔG). (B) The overlap of the predicted tertiary structures (in red) and the corresponding experimentally resolved structures downloaded from the PDBe (in blue), and the RMSD values. (C) Molecular docking models deposited experimentally (in blue) in the PDBe database and the in silico docking models predicted through the described workflow (in red). The target molecules (in green) were isolated from the aptamer-target complexes determined experimentally using the PyMOL software and used as a receptor in the molecular docking prediction. (TIF) [file pone.0264701.s001.tif]

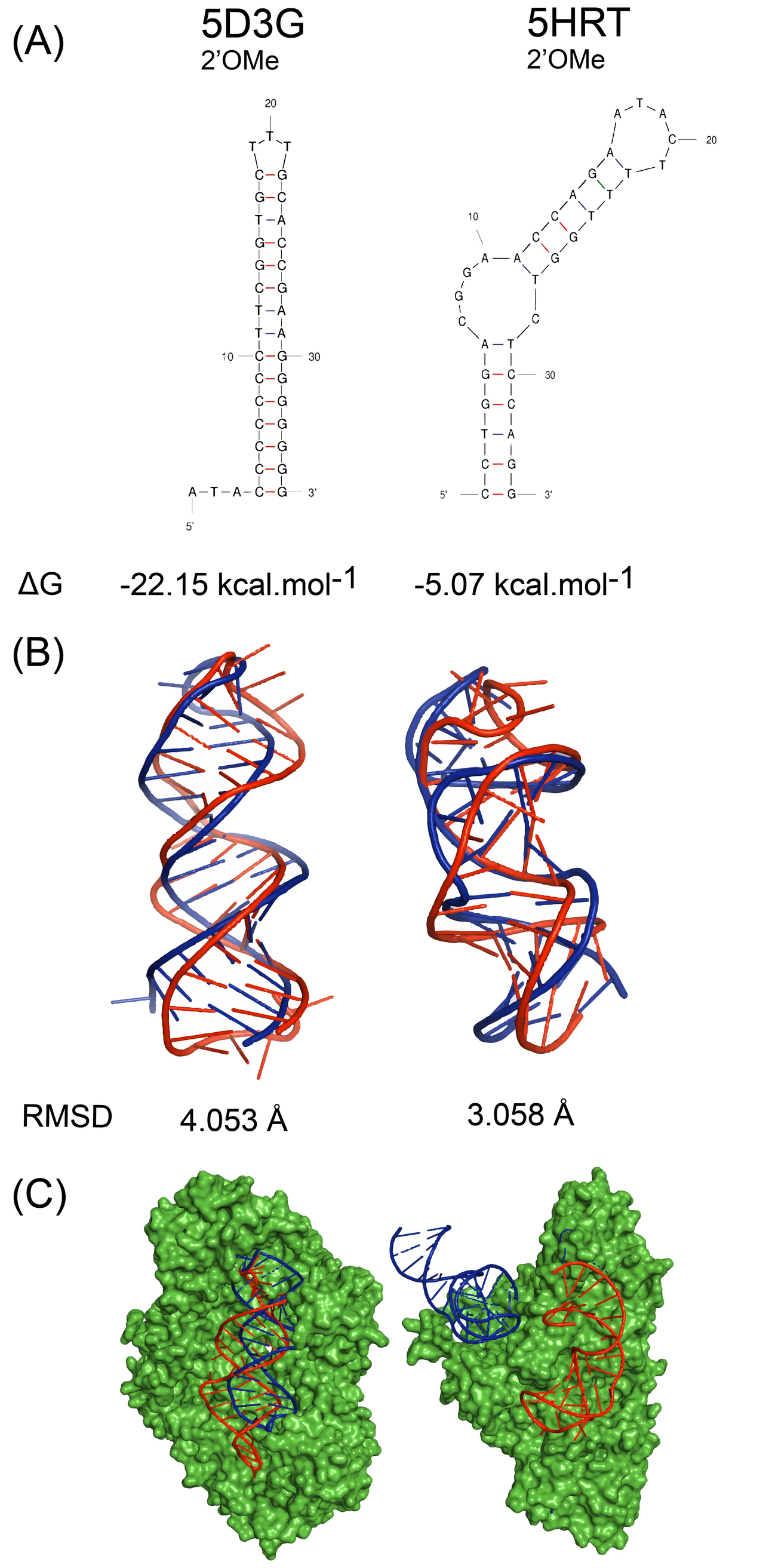

Supplement: S2 Fig — (A) The secondary structures obtained by Mfold and the Gibbs free energy (ΔG) using the corresponding environmental conditions. (B) The overlap of the predicted tertiary structures (in red) and the corresponding experimentally resolved structures downloaded from the PDBe (in blue), and the RMSD values. (C) Docking models deposited experimentally (in blue) in the PDBe database and the in silico docking models predicted through the described workflow (in red). The target molecules (in green) were isolated from the aptamer-target complexes determined experimentally using the PyMOL software and used as a receptor in the docking prediction. (TIF) [file pone.0264701.s002.tif]

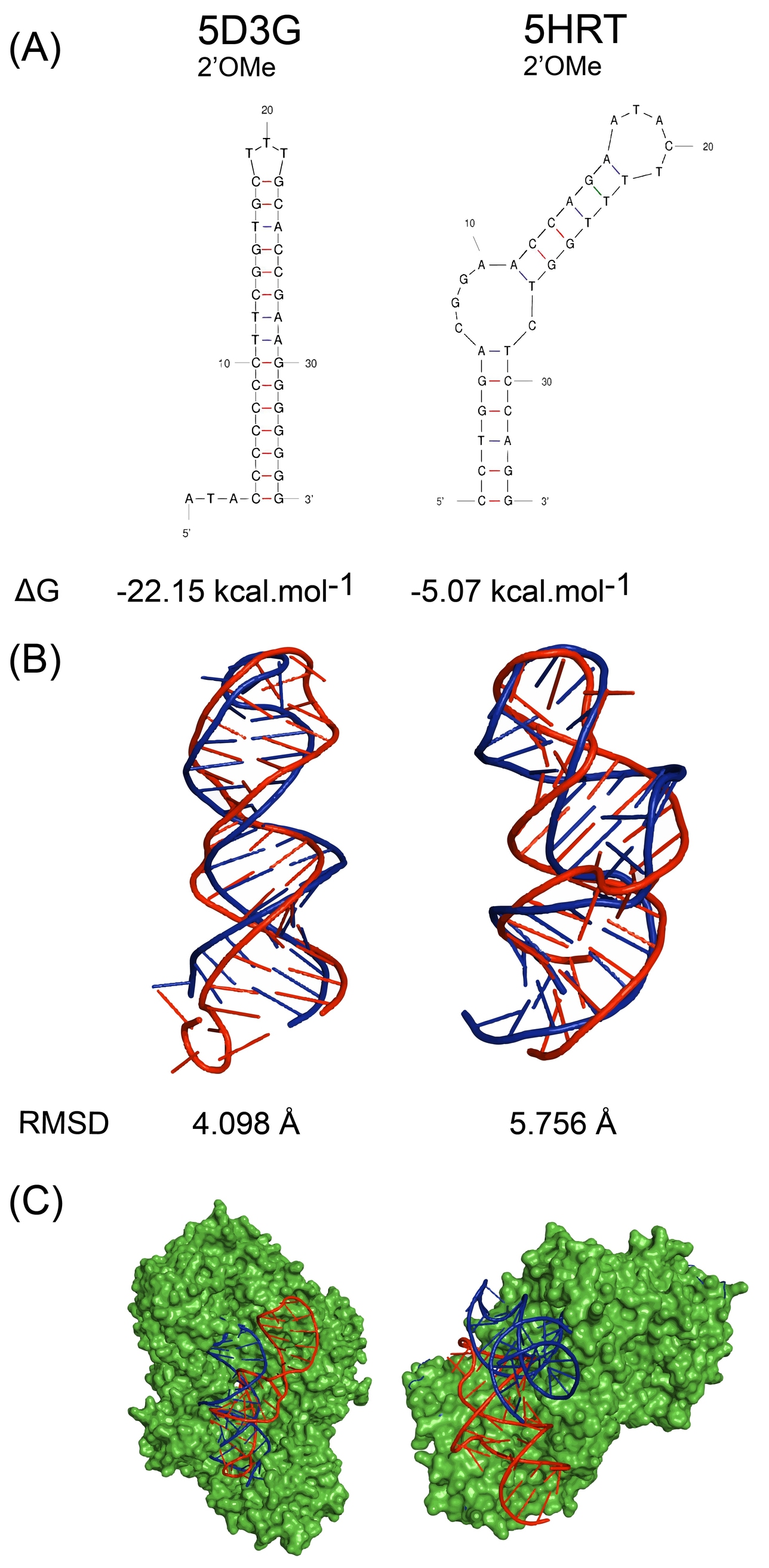

Supplement: S3 Fig — (A) The secondary structures obtained by Mfold and the Gibbs free energy (ΔG) using the corresponding environmental conditions. (B) The overlap of the predicted tertiary structures (in red) and the corresponding experimentally resolved structures downloaded from the PDBe (in blue), and the RMSD values. (C) Docking models deposited experimentally (in blue) in the PDBe database and the in silico docking models predicted through the described workflow (in red). The target molecules (in green) were isolated from the aptamer-target complexes determined experimentally using the PyMOL software and used as a receptor in the docking prediction. (TIF) [file pone.0264701.s003.tif]

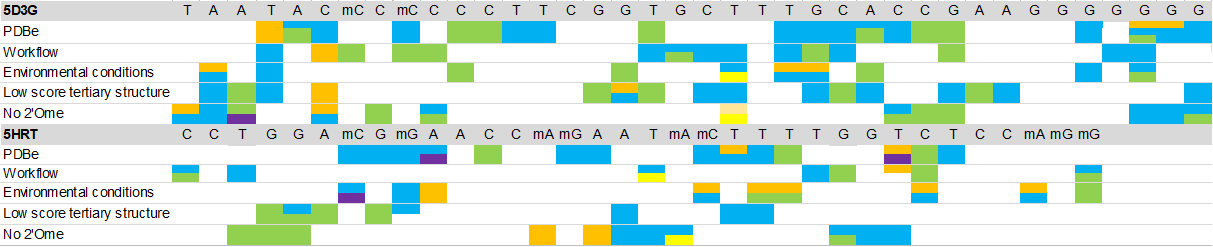

Supplement: S4 Fig — Hydrophobic Interactions (orange), Hydrogen Bonds (blue), Salt Bridges (green), π-Stacking (purple) and π-Cation Interactions (yellow). (TIF) [file pone.0264701.s004.tif]
